# Studying Contextual and Psychological Predictors of Physical Activity Among Emerging Adults: Protocol for an Ecological Momentary Assessment Study

**DOI:** 10.2196/87510

**Published:** 2026-05-06

**Authors:** Sheereen M Harris, Ryan Chen, Calli Naish, Matthew Bourke, Steven R Bray, Matthew Y W Kwan

**Affiliations:** 1 Department of Kinesiology and Health Sciences, University of Waterloo, 200 University Ave. West, LHS 1632, Waterloo, ON, N2L 3G1, Canada, 1 519-888-4567 ext 42252; 2 Department of Community Health Sciences, University of Calgary, Calgary, AB, Canada; 3 Institute for Physical Activity and Nutrition, Deakin University, Geelong, Victoria, Australia; 4 Department of Kinesiology, McMaster University, Hamilton, ON, Canada; 5 Department of Child and Youth Studies, Brock University, St. Catherines, ON, Canada; 6 Health and Wellbeing Centre for Research Innovation, School of Human Movement and Nutrition Sciences, University of Queensland, St Lucia, Queensland, Australia

**Keywords:** exercise, intensive longitudinal data, experience sampling, mental fatigue, young adults

## Abstract

**Background:**

Many adults are insufficiently active, posing a threat to public health. Research shows steep declines in physical activity during the emerging adulthood period. Psychological and socioenvironmental factors have been shown to be independently associated with engaging in physical activity. However, few studies have examined the cross-domain interactions between psychological and socioenvironmental factors on physical activity in real time. Real-time data collection methods can be leveraged alongside traditional nomothetic methods to gain a more comprehensive understanding of how physical activity is affected by dynamic changes to one’s unique psychological and contextual state within a day.

**Objective:**

This paper describes the protocol for a study aiming to examine independent and interactive associations between psychological and contextual factors and real-time physical activity in emerging adults. Ecological momentary assessment (EMA) and device-based monitoring of physical activity will be used.

**Methods:**

The SCOPE (Studying COntextual and Psychological predictors of physical activity among Emerging adults) study will use an intensive longitudinal study design. A total of 124 emerging adults will be recruited, completing 2 waves of data collection consisting of an online survey followed by 7 days of EMAs 6 months apart. EMA surveys will be administered using the Pathverse smartphone app, and physical activity will be assessed using a Fitbit Versa 4 activity monitor.

**Results:**

The study was approved by the research ethics board at the University of Waterloo in November 2025. Recruitment and enrollment began in December 2025, and complete T1 data from 69 participants have been collected as of February 2026. It is expected that T1 data collection will be completed by April 2026, and T2 data collection will occur between June and October 2026. Data analysis on T1 data is expected to begin in May 2026. We anticipate results to be published in fall 2027.

**Conclusions:**

Exploring real-time associations between psychological and socioenvironmental factors and physical activity will provide a more comprehensive understanding of the dynamic barriers and facilitators influencing individuals’ engagement in physical activity in their everyday lives. The outcomes of this work will help advance existing theories on behavioral choice and effort minimization and inform the development of decision rules for adaptive interventions that are tailored to individuals’ unique and current context.

## Introduction

Current public health guidelines recommend adults to engage in a minimum of 150 minutes of moderate-to-vigorous–intensity physical activity (MVPA) weekly to accrue many important health benefits [1,2]. Recent estimates suggest over 30% of adults worldwide do not engage in sufficient amounts of MVPA [3], with evidence indicating higher rates of physical inactivity in higher-income countries [4]. In Canada, recent estimates show that 50% of adults are not meeting current recommendations for MVPA, and 65% of adults were achieving fewer than 7500 steps per day [5]. Trends show that the rates of physical inactivity vary across the lifespan, with evidence of declining MVPA and energy expenditure during the transition from adolescence to adulthood [6-8]. Specifically, the period known as emerging adulthood describes the years between the late teens to mid- or late-20s, with evidence that this period is theoretically and empirically distinct from both adolescence and adulthood [9]. For example, during emerging adulthood, people begin to experience some characteristics traditionally associated with adulthood, such as greater autonomy; however, they have not yet reached important milestones associated with adulthood, such as full-time employment. Prior work examining barriers to engaging in MVPA among emerging adults has identified factors, including stress, perceived skill, peer influences, and class schedules, affecting involvement in physical activity [10]. This demonstrates the important role of psychological, social, and environmental predictors on engagement in physical activity during emerging adulthood and highlights a need to identify behavioral targets for interventions promoting physical activity for this population.

Ecological momentary assessment (EMA) is a real-time data capture methodology involving repeated sampling of people’s current experiences using digital software applications to capture people’s experiences in real time [11]. EMA methods minimize recall bias and maximize ecological validity to better account for the complex and dynamic patterns of change that occur in real-world, real-time scenarios [12]. An additional strength of EMA lies in its potential integration with real-time data collection of physical activity using wearable sensors (eg, accelerometers within smartwatches) to examine associations between psychological states, contextual states, and physical activity in real time.

This study is grounded in socioecological frameworks and dual-process theories. Broadly, socioecological frameworks recognize that behavior is affected by influences from various levels, spanning from psychological states at the individual level to broader social influences, such as public policy [13-15]. According to these frameworks, behavior is shaped not only by the independent effects of factors within different levels but also by the interactions between levels, such as between a person and their environment. Bronfenbrenner’s ecological systems model [13] theorizes the microsystem at the core of the ecological environment, which includes influences from one’s immediate settings and interpersonal relationships, such as face-to-face interactions with family and friends. As an extension of the ecological systems model, the Ecological Model of Physical Activity [16] posits that one’s environment exerts direct and indirect effects on physical activity, highlighting the potential influence of extraindividual factors on physical activity. More broadly, intraindividual and extraindividual influences of behavior can be classified across intrapersonal (eg, personal characteristics and attitudes), interpersonal (eg, formal and informal social networks), institutional (eg, social institutions with shared characteristics), community (eg, relationships among organizations and institutions), and public policy (eg, local and national) levels [14]. This conceptualization highlights the role of both individual-level as well as socioenvironmental factors on health behavior, such as physical activity and the value of assessing influences from multiple levels or domains.

The theory of effort minimization in physical activity (TEMPA) [17] is a dual-process theory, which posits that modern humans have an innate drive to avoid unnecessary physical effort. According to the TEMPA, determinants of physical activity can be characterized as either *controlled* (ie, reflective) processes consisting of slower, deliberate processes and *automatic* (ie, reflexive) processes that are faster, more spontaneous, and automatic by nature. The TEMPA suggests that the perceived effort demands of engaging in physical activity influence both controlled and automatic processes and lead to an effort minimization process in favor of decisions to engage in less effortful behaviors. According to the TEMPA, feelings of fatigue can increase perceptions of behavioral costs, further supporting the effort minimization process. Fatigue has been cited as a common barrier to engaging in regular physical activity [18,19], such that people have a stronger preference for less effortful behaviors under conditions of higher fatigue [20]. Mental fatigue, specifically, refers to a psychobiological state experienced during or following prolonged and challenging cognitive activities [21] and has been shown to bias people’s decisions away from physical activity and toward lower-effort behavioral options [22-24]. However, studies often rely on experimental research in controlled settings or survey-based methods using hypothetical decision-making paradigms with limited generalizability to choices made in naturalistic environments.

Using EMA, 1 study monitoring mental fatigue in people’s daily lives demonstrated that when people reported experiencing higher mental fatigue than their average level, they engaged in fewer minutes of MVPA within the following hours [25]. While these findings provide evidence that psychological factors affect choices to engage in physical activity, examining individual-level factors such as mental fatigue in isolation does not account for people being actors within broader environmental or contextual systems [13,14]. Consistent with socioecological perspectives, the TEMPA acknowledges that effort minimization varies based on characteristics of the individual, behavior, and environment [17]. Previous research has identified associations between psychosocial and socioenvironmental variables, including perceived peer physical activity and family support with MVPA among emerging adults [26]. However, research relying on survey-based data collection methods is unable to capture dynamic changes in psychological states and environmental contexts on a within-day scale. Furthermore, studies examining the cross-domain interactions between psychological and contextual determinants on physical activity in real time are warranted.

Existing EMA research has highlighted associations between social and physical contexts with physical activity [21,27-30]. For example, one study found that 30% and 25% of physical activity observations occurred when adolescents were at school and with classmates [27], respectively. However, the physical and social contexts frequented by younger demographics may differ from those of emerging adults. Specifically, emerging adults’ schedules are often less structured than those of children and adolescents, and additional physical and social contexts not typical in younger groups (eg, postsecondary education, part-time, or full-time employment) might be more common in this demographic. Research in adults revealed that adults engaged in fewer MVPA minutes when at home indoors compared to other physical contexts, and no differences in MVPA were observed based on social context in adults with an age range between 27 and 73 years [29]. A review of 29 EMA studies examining external contexts and movement behavior revealed that greater physical activity was associated with people reporting being outdoors (rather than indoors) and with other people (rather than alone) [30]. However, of the studies included in the review with adult populations, the lowest reported mean age was 38 years (SD=8), which may not be representative of an emerging adult population. Furthermore, despite prior work demonstrating that within-day factors, such as higher positive affect [31] and lower fatigue [25,31], predict greater amounts of subsequent physical activity behavior in adults, few studies have examined cross-domain interactions between psychological and contextual factors on within-day physical activity.

This protocol paper describes the proposed procedures and methodologies for a study aimed at examining psychological and contextual factors predicting real-time physical activity in emerging adults. EMA methodology will be used to capture current psychological states and socioenvironmental contexts, and physical activity will be assessed with device-based passive monitoring. Specific research questions to be addressed in this study include the following:

1. Are there interactive effects between mental fatigue and socioenvironmental contexts on real-time physical activity?
2. What is the effect of anticipated effort on real-time physical activity decision-making?
3. Do controlled and automatic factors associated with physical activity change over time?

## Methods

### Study Design

This study will be an intensive longitudinal study using EMA. Data collection will occur at 2 timepoints (T1 and T2) to account for seasonal variations in physical activity. T1 will take place at the time of enrollment, and T2 will occur 6 months later. At both time points, participants will complete an online survey on Qualtrics followed by EMA surveys distributed 4 times daily for 7 consecutive days (up to 28 total surveys at each timepoint) delivered through the Pathverse app [32]. Pathverse will be used to collect, integrate, and manage study data. EMA data will be collected directly through Pathverse, whereas wearable data processed via Google Health algorithms will be securely extracted and harmonized with the platform. Physical activity will be assessed using a wrist-worn Fitbit Versa 4 activity monitor. To account for differences in sleep schedules, participants will choose between preselected 12-hour waking periods for their prompt schedule, and surveys will be delivered randomly within 4 preprogrammed time windows within participants’ waking period. Participants will be alerted when a survey is available through a notification on their smartphone from the Pathverse app. Each survey will be available for 30 minutes after the initial prompt before closing. The adapted STROBE Checklist for Reporting EMA Studies (CREMAS) is provided in Checklist 1 [33].

### Participants, Recruitment, and Retention

Emerging adults between 18 and 29 years of age living in Southern Ontario, who can communicate in English and own a personal smartphone device with an active data plan, will be recruited. Exclusion criteria will include people with a health issue preventing regular physical activity, people unable or unwilling to wear smartwatches, unable or unwilling to download the Pathverse smartphone app, unable or unwilling to complete daily smartphone prompts during the day, and unable or unwilling to attend 2 in-person measurement visits at the University of Waterloo separated by 6 months. Strategies for participant recruitment will include advertisements in local library branches, local newspapers, the University of Waterloo campus, and social media.

Low compliance is an anticipated challenge of the proposed research due to the potentially burdensome nature of intensive longitudinal data collection [34]. To address potential challenges, first, physical activity will be assessed using provided commercial activity trackers, which are popular among emerging adults [35]. Second, to minimize participant burden during data collection periods, the number of prompts will be limited to a maximum of 4 per day, with surveys requiring 2 to 3 minutes to complete (as determined through pilot testing). Third, the research team will monitor compliance and send a reminder email at the midway point of the data collection period with a summary of survey completion rates and encourage continued participant engagement. A reminder email will also be sent from study researchers to participants, with compliance <50% after the first study day. Fourth, the use of the Pathverse app to administer EMA surveys will send push notifications directly to participants’ personal smartphone device. In addition, participants will be scheduled for their T2 data collection period following the T1 collection period and sent a reminder email. Finally, the incentive structure of the study is designed to promote high compliance, as remuneration will be based on the number of prompts participants respond to, with additional financial incentives for participants achieving high compliance rates (ie, >75% at T1 and T2). In summary, the idea is to reduce participant burden, in that we aim to help push brief EMA prompts to their personal smartphones while using commercial wearables to passively sense behaviors.

### Sample Size

Power was calculated using simulated multilevel data of the hypothesized effect to determine our sample size. Based on an α value of .05, a small interaction effect size between mental fatigue and physical/social context, and 39 observations per participant between T1 and T2 (estimated 70% compliance with EMA surveys based on previous EMA work led by the research team [25]), power calculations using the “power simmixed” program in STATA (StataCorp LLC) indicated a sample size of 103 to achieve 80% power. Based on an expected 20% attrition rate from T1 to T2, 124 participants will be recruited.

### Procedures

Interested individuals will be provided with the study letter of information and asked to confirm their eligibility with a study researcher through email correspondence. Eligible participants will be asked to schedule an in-person orientation session with a study researcher. During the orientation session, a study researcher will review the letter of information and answer any questions from the participant. Next, participants will provide electronic consent prior to completing baseline measures programmed through the Qualtrics survey platform (see *Measures* section).

Next, participants will be familiarized with the study procedures, including the different measures they will respond to during each EMA survey prompt, and be provided with a wrist-worn Fitbit Versa 4 activity monitor, charger, and study reference document. Study researchers will then provide instructions and assistance for downloading the Fitbit and Pathverse smartphone applications onto their personal smartphone device and connecting both apps to the Fitbit device. The Pathverse app used for administering EMA surveys to participants’ personal smartphone devices will have a customized study platform developed by study researchers, and participants will be provided with a unique username and password to activate a secure, personal account and sync with their Fitbit account. Participants will complete 1 practice prompt during the orientation session as a familiarization to the different question types used in the EMA survey. Participants will be given an activity monitor log to record any waking times during which they were not wearing their activity monitor, with a short explanation. A reference document will be provided to each participant, including a summary of the EMA questions, instructions to wear the activity monitor on their nondominant wrist for as much time as they can over the next 7 days, except for showering and charging, and common troubleshooting instructions for the device and accompanying smartphone apps.

The study period will begin the day after the orientation session, and participants will receive prompts to complete the EMA survey 4 times each day for 7 consecutive days. During the orientation period, participants will select one of 3 EMA prompt schedules based on their self-reported typical wake time. Specifically, participants will report usual wake times from 3 options: 7 AM, 8 AM, or 9 AM, with the first survey prompt being sent a minimum of 1 hour following their selected wake time. Surveys will be delivered at random times within 4 preprogrammed 90-minute time windows, each separated by 2 hours: schedule (1) 8:00-9:30 AM, 11:30 AM-1:00 PM, 3:00-4:30 PM, and 6:30-8:00 PM; schedule (2) 9:00-10:30 AM, 12:30-2:00 PM, 4:00-5:30 PM, and 7:30-9:00 PM; and schedule (3) 10:00-11:30 AM, 1:30-3:00 PM, 5:00-6:30 PM, and 8:30-10:00 PM. Participants will be alerted when a survey is available through a notification on their smartphone from the Pathverse app. Following the T1 study period, participants will return the Fitbit device to study researchers and receive their honorarium. At the same time, they will schedule a day/time to return to the laboratory before the T2 collection period approximately 6 months following T1.

Study procedures for T2 are identical to T1 except for offering an abbreviated orientation session as participants have some familiarity with the study procedures, smartphone apps, and Fitbit device. One week prior to their scheduled T2 orientation session, participants will be sent a reminder email and provided with a hyperlink to an online questionnaire programmed through Qualtrics to complete baseline measures prior to beginning the EMA protocol.

### Ethical Considerations

The research ethics application has been reviewed and approved by the University of Waterloo Research Ethics Board (47778). Participants will provide informed consent prior to completing the baseline survey. They will receive CAD $2 (US $1.50) for each EMA survey completed. To encourage participants to complete as many EMA surveys as they can to increase compliance, participants will be offered a CAD $15 (US $11) bonus for completing ≥75% of the surveys during each wave of data collection. Thus, participants can earn up to CAD $142 (US $104) for participation in this study (CAD $71 [US $52] at each of T1 and T2).

Each participant will be given a unique ID used to link their Qualtrics data to their Pathverse data. Participants will also be provided with a unique, deidentified, confidential login and password to access the Pathverse app. Pathverse is HIPAA (Health Insurance Portability and Accountability Act) and PIPEDA (Personal Information Protection and Electronic Documents Act) [36] compliant, and all data will be stored within a Canadian server. The master list linking participants’ initials, email address, and unique participant ID will be stored in an independent password-protected data file (not linked to the study data) on a secure OneDrive server at the University of Waterloo protected using multifactor authentication. The master list will be retained until the second wave of data collection is completed before being permanently deleted. Baseline survey, EMA data, and activity data will be deidentified and stored on an electronic spreadsheet on a secure OneDrive server. Data from Qualtrics and Pathverse will be stored in a secure file accessed only by members of the research team. Data will be maintained on file for a minimum of 5 years after publication.

### Measures

#### EMA Measures

Participants will respond to up to 4 EMAs each day over the 7-day study period at both T1 and T2. To minimize participant burden, very brief surveys were developed, designed to take approximately 2 minutes to complete.

##### Self-Reported Sleep Duration

During the first EMA survey of each day, participants will be asked to self-report their sleep duration from the prior night. Using a self-made item, participants will respond to the question: “How many hours of sleep did you get in total last night?” Responses will be provided using a drop-down menu from 0 to 12+ hours, increasing in 0.5 hour increments.

##### Self-Reported Physical Activity

Participants will be asked to self-report the minutes of physical activity they have engaged in during different time periods. The first survey for each day will ask the following 2 questions: “Since responding to the last survey yesterday, did you engage in any moderate-to-vigorous–intensity physical activity yesterday?” and “Before the first survey for today, did you engage in any moderate-to-vigorous–intensity physical activity?” The remaining prompts each day will ask participants: “Since the last survey, did you engage in any moderate-to-vigorous–intensity physical activity?” Each survey will provide examples of MVPA (eg, brisk walking, cycling, running, and aerobics). Response options will be as follows: “No (less than 10 min),” “Yes (10-19 min),” “Yes (20-29 min),” “Yes (30-39 min),” “Yes (40-49 min),” “Yes (50-59 min),” “Yes (60-69 min),” and “Yes (70+ min).” The wordings for these items were adapted from previous EMA research assessing self-reported physical activity in the past 24 hours [37].

##### Current Activity

Participants will be asked to self-report their current activity. Participants will respond to the following question: “What were you doing right before you opened this prompt.” Response options are based on prior EMA research [38] and feedback from pilot testing to include physical activity or exercise, reading/studying or doing homework, leisure or social activities, working or at your job, on a computer, phone, or tablet for personal use, playing computer/video games, watching TV or a movie, eating a meal, housework or chores, or other. Participants indicating that they were engaging in physical activity/exercise or “other” activity will be prompted to describe their current activity in freeform text.

##### Mental Fatigue

Based on prior EMA research [25], a 101-point horizontal sliding scale, similar to a Visual Analog Scale [39], will be used to assess mental fatigue. Participants will be instructed to: “Please use the sliding scale to indicate what you feel represents your perception of your current state of mental fatigue” with the anchors ranging from “None at all” (0) on the far left to “Maximal” (100) on the far right.

##### Mood

Mood will be assessed using the Short Mood Scale to assess the dimensions of valence, energy, and calmness [40]. Participants will respond to the statement: “At this moment I feel:” and respond to the 6 paired statements of tired-awake (energy), content-discontent (valence), agitated-calm (calmness), full of energy-without energy (energy), unwell-well (valence), and relaxed-tense (calmness). Response options range from 0 to 6, with the label “very” at both end points. Responses from content-discontent, full of energy-without energy, and relaxed-tense will be reverse-scored, and a mean score will be computed. Within-person reliability has been reported as 0.70 for valence and calmness and 0.77 for arousal. Structural validity of the 3-dimensional structure at the within-person level has been reported [41].

##### Short-Term Behavioral Intention

Modeling an approach used previously in EMA research [42,43], short-term physical activity intention will be assessed using a single-item measure. Participants will respond to the question: “I intend to be physically active for 10+ minutes sometime within the next 60 minutes,” with response options scored on a 7-point scale ranging from (1) strongly disagree to (7) strongly agree.

##### Anticipated Effort

A 101-point horizontal sliding scale, similar to a Visual Analog Scale [39], will be used to assess anticipated effort. Participants will be asked: “Within the next hour, how much effort do you feel it will take to get ready to engage in physical activity,” with anchors ranging from “No effort at all” (0) on the far left to “Very maximal effort” (100) on the far right. This item was created based on previous non-EMA research examining anticipated effort [23].

##### Physical Context and Perceived Quality

Response options for physical context and perceived quality are based on prior EMA research assessing contexts in adults’ daily lives [29]. Participants will report their current location at the time of the survey prompt from the following response options: home—indoors (eg, bedroom, living room, and kitchen), home—outdoors (eg, balcony, yard, and driveway), work (indoors), school (indoors), not at home—outdoors (eg, park, trail, sidewalk, and road), car/van/truck.

Based on prior EMA research [29], perceived quality of outdoor physical locations, including perceived safety, greenness/vegetation, traffic, and litter, will be assessed: “How safe do you feel right now?” with response options on a 5-point scale ranging from “Not at all” (1) to “Very much so” (5). Participants will respond to the question: “How many trees and plants are there in the area where you are right now?” with response options of Not applicable—currently indoors, no trees or plants, a few trees and plants, some trees and plants, and a lot of trees and plants. Participants will respond to the question: “How much traffic is on the closest street to where you are right now?” with response options of Not applicable—currently indoors, no traffic, a little traffic, some traffic, and a lot of traffic. Participants will respond to the question: “How much litter or garbage is on the ground where you are right now?” with response options of Not applicable—currently indoors, no litter, a little litter, some litter, and a lot of litter.

##### Social Context and Perceived Quality

Response options for physical context are based on prior EMA research assessing contexts in adults’ daily lives [29]. Participants will first report their current social context at the time of the survey prompt from the following response options: By myself, with spouse or partner, with child or children, with other family members, with friends, with coworkers, with other types of acquaintances, and with people I do not know.

Based on previous EMA research [44], perceived quality of social context will be assessed using a single-item measure assessing each of familiarity and importance. Participants will respond to the question: “How well do you know this person/these people?” with response options presented on a horizontal sliding scale, similar to a Visual Analog Scale [39], ranging from “Not at all” (0) to “Very well” (100)*.* Participants will then respond to the question: *“*How important was this interaction for you?” with response options presented on a horizontal sliding scale, similar to a Visual Analog Scale [39], ranging from “Not at all” (0) to “Very important” (100)*.*

### Baseline Measures

The following measures will be assessed using an online questionnaire administered at baseline (T1) and 6-month follow-up (T2).

#### Demographic factors

Participants will be asked to respond to demographic questions, including self-reporting age, gender, race, and year of study/education level.

#### Self-Reported Weekly Physical Activity

Four items from the International Physical Activity Questionnaire [45] will be used to assess weekly MVPA. Participants will report how many days per week and minutes per day they spent engaging in each of MVPA during the past 7 days.

#### Physical Activity Enjoyment

The Physical Activity Enjoyment Scale (PACES) [46] will be used to assess physical activity enjoyment. Consistent with other studies [47], the wording of the original scale will be altered from “Please rate how you feel at the moment about the physical activity you have been doing” to “Please rate how you feel while engaging in physical activity” to represent participants’ overall enjoyment of physical activity. The PACES consists of 18 paired statements (eg, “I enjoy it”—“I hate it”) scored on 7-point semantic differential scales. A composite score will be computed according to the instrument’s instructions.

#### Approach Tendencies Toward Physical Effort

The Physical Effort Scale (PES) [48] will be used to assess one’s tendency to approach and to avoid physical effort. The PES consists of 4 statements assessing the *approach* dimension of physical effort (eg, “The idea of exerting physical effort usually appeals to me”) and 4 statements assessing the *avoidance* dimension of physical effort (eg, “I usually dislike activities that involve physical effort”). Items will be scored on a 5-point scale ranging from “I completely disagree” (1) to “Completely agree” (5). Consistent with instrument instructions, separate scores will be computed for *Approach* and *Avoidance* tendencies toward effort separately, as well as a relative tendency to approach rather than avoid (mean *Approach* score—mean *Avoidance* score).

#### Physical Activity Attitudes

Six items will be used to assess instrumental and affective attitudes for engaging in physical activity [49]. The instrumental items are wise, beneficial, and useful, and the affective items are enjoyable, exciting, and pleasant. The statement proceeding the items will be: “Over the next 7 days, engaging in physical activity on a regular basis would be…” and scored on a 7-point scale ranging from “Extremely disagree” (1) to “Extremely agree” (7)*.* The mean score of all items will be computed.

#### Perceived Capability

Three items will be used to assess perceived capability over physical activity [50,51]. The items are: “I possess the skills to do regular physical activity over the next 7 days if I wanted to,” “I have the physical ability to do regular physical activity over the next 7 days if I wanted to,” and “I am confident that I am capable of engaging in regular physical activity if I had to.” Items will be scored on a 5-point scale ranging from “Strongly disagree” (1) to “Strongly agree” (5)*.* The mean score of all items will be computed.

#### Perceived Opportunity

Three items will be used to assess perceived opportunity for physical activity [50,51]. The items are: “If I really wanted to do regular physical activity over the next 7 days, I would have the chance to do so,” “I lack the opportunity to do regular physical activity over the next 7 days, even if I were really motivated to do so,” and “There are places where I can do physical activity at home and at work if I had to.” Items will be scored on a 5-point scale ranging from “Strongly disagree” (1) to “Strongly agree” (5)*.* Responses from the second item will be reverse-scored, and a mean score of all items will be computed.

#### Physical Activity Intentions

One item will be used to assess intentions to be physically active. Participants will be asked to indicate the number of times they intend to engage in MVPA during the next 7 days [52].

#### Habit

The Self-Report Behavioral Automaticity Index (SRBAI) [53] will be used to assess habit. The scale consists of 4 statements rated on a 7-point scale ranging from “Strongly disagree” (1) to “Strongly agree” (7). The items are: “I do automatically,” “I do without having to consciously remember,” “I do without thinking,” and “I start doing before I realize I’m doing it.” Each item will be preceded by the statement: “Regular physical activity is something….” A composite score will be computed according to the instrument instructions.

#### Physical Activity Identity

Identity will be assessed using 4 items [54,55] and the Multidimensional Inventory of Physical Activity Identity (MIPAI-25) [56]. The items are: “I consider myself someone who does regular physical activity,” “When I describe myself to others, I usually include my involvement in physical activity,” “Others see me as someone who does physical activity regularly,” and “Regular physical activity fits the way I want to live.” These items are rated on a 5-point scale ranging from “Strongly disagree” (1) to “Strongly agree” (5)*.* The mean score of all items will be computed.

The MIPAI consists of 25 statements across the dimensions of *acknowledgment* (eg, “Compared to others my age, I am a physically active individual”), *compatibility* (“eg, Being physically active is something that fits the way I want to live”), *centrality* (eg, “I see myself as a physically active person”), *commitment* (eg, “I am committed toward being physically active”), and *exploration* (eg, “I try to find out a lot about physical activity”). Each item will be scored on a 7-point scale ranging from “Strongly disagree” (1) to “Strongly agree” (7), and the mean overall score will be computed.

#### Behavioral Regulation

In addition to examining the influence of reflective and reflexive processes, this study will also look to explore the potential role of the regulatory process on physical activity. Regulatory processes, including behavioral regulation, are theorized to help translate intentions into behavior until reflexive processes allow for behavior to occur automatically [57]. Behavioral regulation for physical activity will be assessed using the Physical Activity Regulation Scale [58]. Fourteen items to identify strategies, including self-monitoring, action planning, and reducing negative emotions, to help engage in physical activity [59,60]. Sample items include: “To be physically active, I have made a detailed plan regarding when to be physically active,” “I record my physical activities,” and “When I am upset, I use strategies to feel better so I can be physically active.” Items will be scored on a 7-point scale ranging from “Strongly disagree” (1) to “Strongly agree” (7)*.* Mean scores will be computed according to the scale instructions for proactive regulation, reactive regulation, social monitoring, and self-monitoring.

### Device-Assessed Physical Activity

Participants will be provided with a wrist-worn Fitbit Versa 4 device and asked to wear it continuously on their nondominant wrist for 7 consecutive days during both data collection waves to passively assess physical activity. Fitbit devices were selected due to compatibility with both iOS and Android-operating software. Research shows, in comparison with criterion measures, wrist-worn Fitbit devices have similar percent bias values compared to research-grade accelerometers for assessing steps (Fitbit Flex: −23%, Actigraph GT3X: −32%) and active minutes (Fitbit Flex: −65%, Actigraph GT3X: −40%), demonstrating a similar degree of error and good agreement in physical activity estimates from these commercially available monitors and Actigraph accelerometers, which are considered the industry standard for research-grade physical activity measurement [61].

Active minutes and steps will be processed using Google Health algorithms, and summary data will be accessed by Pathverse through the Fitbit application programming interface. Active minutes (lightly active, fairly active, very active, and sedentary) will be calculated through metabolic equivalents (METs) to estimate exercise intensity through a ratio of rate of energy expended during an activity/rate of energy expended during rest. Active minutes for non–step-based activities (eg, weight lifting and yoga) are calculated using heart rate [62]. Intensity classifications are based on proprietary MET-based algorithms; specific MET thresholds are not publicly disclosed. MVPA minutes will be operationalized as the sum of fairly active and very active minutes. Steps will be calculated using the device’s 3-axis accelerometer. Device wear time will be calculated using minute-level heart rate data, with valid wear defined as nonzero heart rate minutes.

### Data Analysis Plan

Descriptive data from the baseline survey will be summarized as means (SD) and frequencies as applicable. Descriptive statistics for EMA surveys will be calculated with observation (ie, EMA prompt) as the unit of analysis. Missing data will be analyzed using logistic regression models. Multilevel mixed effects linear models will be computed to examine associations between EMA variables and physical activity according to study aims. In each analysis, between-person effects (ie, how each participant’s scores differed from those of other participants) and within-person effects (ie, how people’s scores fluctuated within themselves across prompts) will be disaggregated using grand mean and person-mean centering, respectively [63]. To answer the first research question, a mixed effects model with the person-mean-centered mental fatigue and socioenvironmental context will be entered as fixed effects; in addition, interaction terms between fatigue and socioenvironmental variables will be included to examine moderation effects. To answer the second research question, mixed effect models to regress physical activity behaviors and decision-making (ie, short-term behavioral intentions) on person-mean-centered anticipated effort will be estimated. To answer the third research question, a repeated cross-sectional analysis will be conducted to determine the association between controlled and automatic predictors of physical activity at each assessment. Additionally, to determine if controlled and automatic predictors are associated with physical activity change over time, a mixed effect model will be estimated with physical activity as the outcome, time as a fixed effect (ie, T1 and T2), controlled and automatic predictors as fixed effects, and an interaction term between time and controlled and automatic predictors. The model will control for demographic covariates in addition to the season of data collection to account for seasonal variation in physical activity. Each model will include a random intercept to account for the correlation among repeated assessments. Additionally, random slopes will be estimated and retained if they significantly improve model fit.

## Results

This study has been funded by the Social Sciences and Humanities Research Council of Canada in June 2025 and the Canada Foundation of Innovation John R. Evans Leaders Fund and Ontario Research Fund—Research Infrastructure in June 2024. Participant recruitment began in December 2025, and complete T1 data from 69 participants have been collected as of February 2026. It is expected that T1 data collection will be completed by April 2026, and T2 data collection will occur between June and October 2026. The data analysis on T1 data is expected to begin in May 2026. We anticipate results to be published in fall 2027.

## Discussion

This study will investigate the independent and interactive influences of psychological and socioenvironmental factors on physical activity in real time among emerging adults. This study addresses a critical need for a nuanced understanding of how dynamic interactions between mental fatigue and social and physical contexts impact effort-based decision-making. Study findings will contribute new information relating to existing theories on effort-based decision-making by considering the dynamic associations between psychological and environmental factors and effort-based choices in real-time, naturalistic environments. This work takes a novel approach to study the impact of predictors of effort-based choice. Specifically, leveraging novel methodologies, such as EMA, will further our understanding of conditions affecting decision-making related to engaging in physical activity and aid the development of interventions promoting decisions to exert effort tailored to an individual’s unique and evolving psychological and contextual state.

We have outlined several strategies to enhance compliance based on recommended strategies for young adults [64], such as email reminders and tailoring schedules to participants’ self-reported wake time. To minimize participant burden, up to 4 survey prompts will be administered daily, with each survey designed to take 2 to 3 minutes to complete based on pilot testing. Based on recommendations from previous work in young adults [64], participants will be consulted to determine an individualized prompt schedule based on their typical sleep schedule. In addition, a reminder email will also be sent from study researchers to participants, with compliance <50% after the first study day, and for all participants midway through the study period with an update on their current compliance and a reminder of incentive bonuses based on overall compliance to promote high compliance. We have accounted for a 20% attrition rate from T1 to T2 in our sample size calculation, anticipating some attrition due to the longitudinal nature of data collection. Finally, the incentive bonuses at both T1 and T2 based on high overall compliance rates were included to encourage engagement with the EMA protocol. The research team is experienced with EMA methods and has successfully used these and other strategies, including the response incentives, in previous projects [25].

In light of evidence demonstrating that adults are not meeting recommended levels of physical activity [3,5], with declines occurring during the emerging adulthood period [6-8], understanding how people make decisions related to their physical activity behaviors is pivotal in ensuring that people maximize opportunities to experience the personal, psychological, and social benefits of physical activity and improved overall well-being. This work will address critical gaps in knowledge regarding effort-based decision-making and inform interventions promoting physical activity behaviors among emerging adults. Overall, study findings will help identify behavioral targets for real-time digital interventions in an emerging adult population. The study will additionally advance theory by testing associations in real-time, dynamic contexts and extend current theories on behavioral choice and effort minimization in physical activity.

## Supplementary material

10.2196/87510 Checklist 1 Adapted STROBE Checklist for Reporting EMA Studies (CREMAS).

## Abbreviations

CREMAS: Checklist for Reporting EMA Studies
EMA: ecological momentary assessment
HIPAA: Health Insurance Portability and Accountability Act
MET: metabolic equivalent
MIPAI-25: Multidimensional Inventory of Physical Activity Identity
MVPA: moderate-to-vigorous intensity physical activity
PACES: Physical Activity Enjoyment Scale
PES: Physical Effort Scale
PIPEDA: Personal Information Protection and Electronic Documents Act
SCOPE: Studying Contextual and Psychological Predictors of Physical Activity Among Emerging Adults
SRBAI: Self-Report Behavioral Automaticity Index
TEMPA: theory of effort minimization in physical activity
